# Overlapping of Genes in the Human Genome

**Published:** 2007-03

**Authors:** Tomohiro Nakayama, Satoshi Asai, Yasuo Takahashi, Oto Maekawa, Yasuji Kasama

**Affiliations:** 1 *Division of Molecular Diagnostics, Advanced Medical Research Center, Nihon University School of Medicine, Tokyo, Japan*; 2 *Division of Genomic Epidemiology & Clinical Trials, Advanced Medical Research Center at the Nihon University School of Medicine, Tokyo, Japan;*; 3 *Maize Corporation, Tokyo, Japan*

**Keywords:** overlapping genes, human genome, locus, exon, chromosome

## Abstract

Overlapping genes are relatively common in DNA and RNA viruses. There are several examples in bacterial and eukaryotic genomes, but, in general, overlapping genes are quite rare in organisms other than viruses. There have been a few reports of overlapping genes in mammalian genomes. The present study identified all of the overlapping loci and overlapping exons in every chromosome of the human genome using a public database. The total number of overlapping loci on the same and opposite strands was 949 and 743, respectively. Similarly, in every chromosome, the instances in which two loci were located on the same strand was similar to the number of 2 genes observed on opposite strands, except for chromosome 5. The number of 2 exons located on the same strand was higher than that for 2 exons located on opposite strands, indicating the presence of many comprehensive-type overlaps. The mean percentage of overlapping exons on opposite strands in each chromosome was 3.3%, suggesting that parts of the nucleotide sequences of 26,501 exons are used to produce 2 transcribed products from each strand. The ratio of the number of overlapping regions to chromosomal length revealed that, on chromosomes 22, 17 and 19, ratios were high for both types of 2 loci, with exons located on the same and opposite strands. Ratios were low on chromosomes Y, 13 and 18. These results show that all overlapping types are distributed throughout the human genome, but that distributions differ for each chromosome.

## INTRODUCTION

Publication of the human genome sequence marked a significant milestone in the field of biology. Significant biological information can also be gained from the analysis of genome organization. Approximately 30,000 protein-coding genes are thought to be present in the human genome, and the positions of genes within chromosomes is currently being established (1-4).

DNA sequences can code for more than one gene product by using different reading frames or different initiation codons. Overlapping genes are relatively common in DNA and RNA viruses (5-9). While several examples exist in bacterial and eukaryotic genomes, overlapping genes appear to be relatively rare in non-viral organisms and few reports have described overlapping genes in mammalian genomes (10-12). Some studies have demonstrated that the overlapping of genes differs among species and have inferred that this can be attributed to differences in evolutionary histories (13-15).

Given that both strands of the human genome are used for transcription, two types of overlapping are thus possible; 2 genes overlapping on the same strand, and 2 genes overlapping on opposing strands. Furthermore, overlapping patterns can be classified by the relative positions of the 2 genes. Little exact information is available regarding overlapping genes in the human genome and their associated overlapping patterns. To investigate this phenomenon and the inherent biological information contained therein further, the number of overlapping genes and their patterns were examined in every chromosome of the human genome.

## METHODS

The positions and sequences of each gene were obtained from the National Center for Biotechnology Information (NCBI) database (build 31; published January 15, 2003; http://www.ncbi.nlm.nih.gov/genome/guide/human/HsStats.html). Each locus was defined using both LocusLink and RefSeq, using gene symbols and names established by the nomenclature committee for the genome (http://www.gene.ucl.ac.uk/nomenclature/).

In the LocusLink report, symbols and names were reported under the banner (http://www.ncbi.nlm.nih.gov/). Exons were defined as DNA sequences coding mRNA, rather than considering functions within specific genes or locations within specific genes. This definition allowed for analysis of all possible cases of overlapping. Official gene symbols and names were used as follows: For RefSeq records, symbols were assigned using the LOCUS system. If a symbol had not yet officially been assigned, an interim symbol and name were arbitrarily selected. Arbitrarily selected symbols and names are included at this website http://www.ncbi.nlm.nih.gov/LocusLink/collaborators.html).

All loci and exons registered in NCBI build 31 were examined, using the data describing the position of genes on the chromosome. The information of nucleotide sequences and the positions of each nucleotide in the whole human genome were downloaded and stored in EXCEL file format (Microsoft Corporation, Redmond, Washington). All overlapping loci and overlapping exons could be defined according to the start and end positions of each locus and exon. Data for overlapping loci were produced using data of registered loci, while the data for overlapping exons was produced using data distinct coding regions and mRNA sequences.

## RESULTS

**Figure 1:**
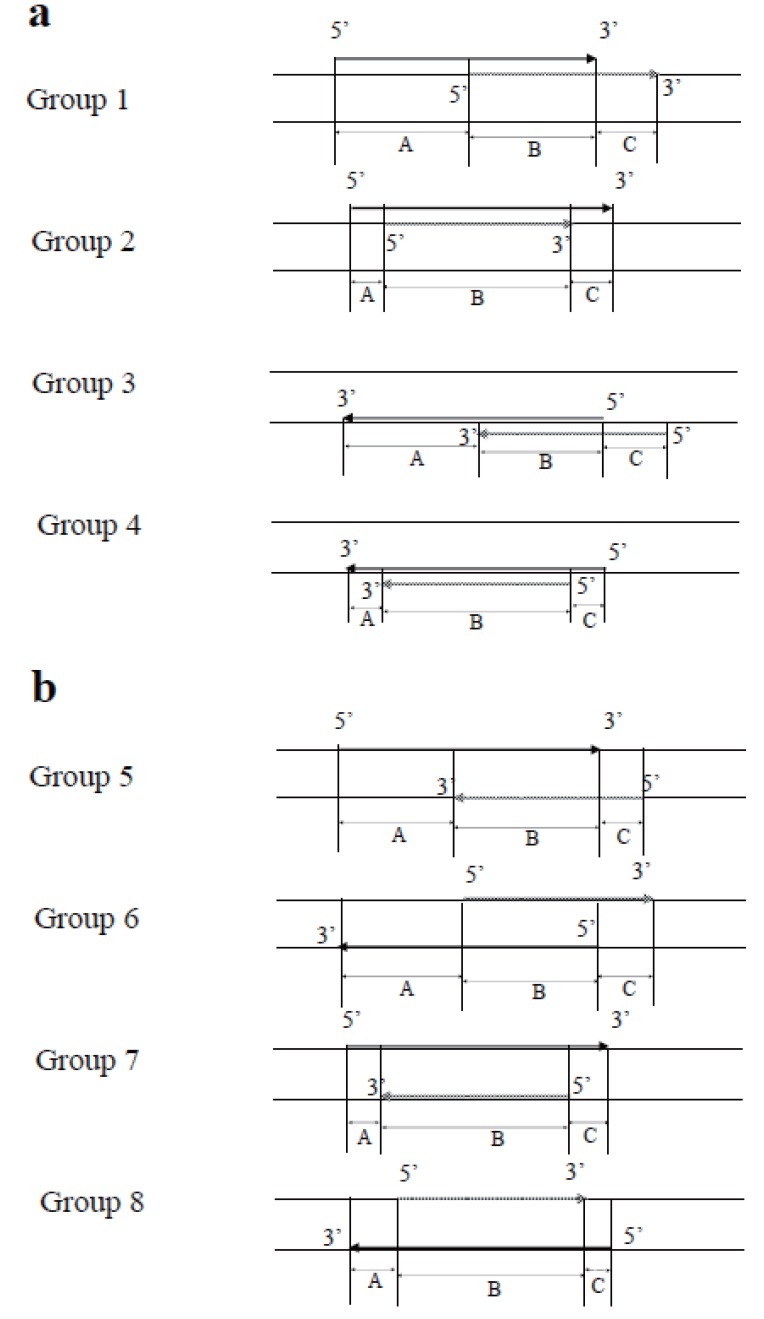
Schema of overlapping patterns among loci or exons. Black arrows indicate locus or exon “1”. Shaded arrows indicate locus or exon “2”. Locus “2” or exon “2” were defined as those with the side of short arm (p) were located downstream of locus 1 or exon 1. A was the length of the flanking region without overlapping, located on the side of short arm (p). B was the length of the overlapping region. C was the length of the flanking region without overlapping, located on the side of long arm (q). a: Schema of 2 loci or exons on the same strand. Groups 1 and 2: both regions are on the positive strand (unidirectional). Groups 3 and 4: both regions are on the negative strand (unidirectional). Groups 2 and 4: region 1 includes region 2 (comprehensive). b: Schema of 2 loci or exons on opposite strands. Group 5: convergent; Group 6: divergent; Groups 7 and 8: comprehensive.

The type of overlap was classified into 8 groups (Fig. 1), with 2 loci or exons on the same strand divided into 4 groups, and the same for those on opposite strands. We classified the patterns of overlapping genes by considering the strand-location of respective genes. For example, for Groups 1 and 2, both regions are on the positive (sense) strand and their mRNAs are transcribed using the negative (antisense) strand as the template. Groups 1, 2 and 7 were also different from Groups 3, 4, and 8, respectively.

General information for overlapping loci is shown in Table 1. A total of 12,692 loci were present on the positive strand, with 12,442 on the negative strand. The total number of overlapping loci on the same and opposite strands was 949 and 743, respectively. Except for Group 2 of chromosome 5, the number of instances where 2 loci were located on the same strand was similar to the number of 2 loci on opposite strands on every chromosome. This group on chromosome 5 includes the protocadherin (PCDH) cluster located on the positive strand of 5q31. The mean number of overlapping loci was 6.7% of the total loci on each chromosome (range, 2.0-33.7%). The ratio of the number of overlapping loci to chromosome length revealed chromosomal characteristics. On chromosomes 22, 17 and 19, ratios were high when 2 loci were on overlapped both the same and opposite strands. Analysis of overlap type revealed Groups 2 and 6 as occurring relatively frequently.

**Table 1:** Number of overlapping loci

Chromosome	Number of loci		Two loci located on same strand							Two exons located on the opposite strand							Total		
	Positive	Negative	Positive-Positive		Negative-Negative		Total	Frequency (%)	Ratio of overlapping loci/chromosome	Positive-Negative Convergent Group 5	Negative-Positive Divergent Group 6	Positive-Negative Comprehension Group 7	Negative-Positive Comprehension Group 8	Total	Frequency (%)	Ratio of overlapping loci/chromosome	Total	Frequency (%)	Ratio of overlapping loci/chromosome
			Unidirection Group 1	Comprehension Group 2	Unidirection Group 3	Comprehension Group 4													

1	1,226	1,178	18	5	21	14	58	2.4	0.26	34	19	8	6	67	2.8	0.30	125	5.2	0.56
2	901	824	9	4	10	5	28	1.6	0.12	20	15	1	2	38	2.2	0.16	66	3.8	0.28
3	688	661	10	2	10	5	27	2.0	0.14	22	11	6	5	44	3.3	0.22	71	5.3	0.36
4	509	527	6	1	3	3	13	1.3	0.07	15	12	2	2	31	3.0	0.17	44	4.2	0.24
5	629	557	10	341	10	4	365	30.8	2.01	17	10	5	3	35	3.0	0.19	400	33.7	2.20
6	640	676	6	1	10	6	23	1.7	0.13	14	12	6	2	34	2.6	0.20	57	4.3	0.33
7	603	580	8	2	13	2	25	2.1	0.17	19	14	2	4	39	3.3	0.27	64	5.4	0.44
8	436	471	6	1	7	5	19	2.1	0.13	10	7	3	3	23	2.5	0.16	42	4.6	0.29
9	489	560	9	4	12	2	27	2.6	0.24	16	8	4	4	32	3.1	0.28	59	5.6	0.52
10	502	472	14	3	15	1	33	3.4	0.25	9	7	2	1	19	2.0	0.15	52	5.3	0.40
11	798	782	5	6	9	6	26	1.6	0.20	22	9	4	6	41	2.6	0.31	67	4.2	0.51
12	616	617	11	6	13	8	38	3.1	0.28	18	9	2	13	42	3.4	0.31	80	6.5	0.59
13	228	258	1	2	5	0	8	1.6	0.08	5	4	3	0	12	2.5	0.12	20	4.1	0.20
14	407	384	8	1	7	0	16	2.0	0.18	11	8	0	1	20	2.5	0.23	36	4.6	0.41
15	402	435	3	2	10	4	19	2.3	0.24	11	9	0	5	25	3.0	0.31	44	5.3	0.55
16	569	471	8	1	9	1	19	1.8	0.25	33	8	1	1	43	4.1	0.57	62	6.0	0.82
17	629	707	11	8	16	9	44	3.3	0.56	33	12	8	5	58	4.3	0.74	102	7.6	1.30
18	217	189	2	1	3	0	6	1.5	0.08	3	4	0	0	7	1.7	0.09	13	3.2	0.17
19	786	750	10	7	13	8	38	2.5	0.66	24	10	1	0	35	2.3	0.60	73	4.8	1.26
20	334	321	6	0	10	5	21	3.2	0.34	10	9	1	6	26	4.0	0.43	47	7.2	0.77
21	150	134	4	7	1	1	13	4.6	0.39	4	1	0	4	9	3.2	0.27	22	7.7	0.66
22	295	298	7	24	6	2	39	6.6	1.08	16	6	18	4	44	7.4	1.22	83	14.0	2.30
X	560	515	21	16	4	1	42	3.9	0.33	5	4	2	7	18	1.7	0.14	60	5.6	0.47
Y	78	75	1	1	0	0	2	1.3	0.11	1	0	0	0	1	0.7	0.05	3	2.0	0.16
Total	12,692	12,442	194	446	217	92	949	3.8	0.33	372	208	79	84	743	3.0	0.26	1692	6.7	0.59

Given that the organization of several genes has not yet been clarified, insufficient information is currently available for determining the incidence/pervasiveness of overlapping loci in these specific genes. We therefore set about to examine overlapping exons for the human genome as a whole.

The total number of exons on the positive and negative strands was 404,776 and 402,510, respectively (Table 2). The number of 2 exons located on the same strand (3,843,308) differed substantially from the number of 2 exons on opposite strands (26,501). Interestingly, the number of 2 exons located on the same strands (groups 2 and 4) exceeded the number of exons, with comprehensive-type overlaps (Groups 7 and 8). This indicates the presence of numerous comprehensive-type overlaps on the same strands (2 exons located on the same strand with a smaller exon within the larger exon). The percentage of overlapping exons (out of the total number of exons) on opposite strands within each chromosome ranged from 1.1% to 5.5%. The total number of overlapping exons was 26,501 (3.3%) out of 807,286 exons, which suggests that parts of the nucleotide sequences for 26,501 exons are used to produce 2 transcribed products from each strand.

**Table 2:** Number of overlapping exons

Chromosome	Number of exons		Two exons located on same strand							Two exons located on the opposite strand							Total		
	Positive	Negative	Positive-Positive		Negative-Negative		Total	Frequency (%)	Ratio of overlapping loci/chromosome	Positive-Negative Convergent Group 5	Negative-Positive Divergent Group 6	Positive-Negative Comprehension Group 7	Negative-Positive Comprehension Group 8	Total	Frequency (%)	Ratio of overlapping loci/chromosome	Total	Frequency (%)	Ratio of overlapping loci/chromosome
			Unidirection Group 1	Comprehension Group 2	Unidirection Group 3	Comprehension Group 4													

1	40,542	40,285	893	168,929	1,020	194,722	365,564	452.3	1,662	383	265	717	794	2,159	2.7	9.81	367,723	455.0	1,671
2	28,527	31,849	1,145	111,580	5,906	167,407	286,038	473.8	1,192	259	240	693	581	1,773	2.9	7.39	287,811	476.7	1,199
3	24,362	23,336	667	117,639	552	87,753	206,610	433.2	1,033	414	175	621	545	1,755	3.7	8.78	208,365	436.8	1,042
4	14,710	14,759	164	52,399	252	56,197	109,012	369.9	586	65	58	369	475	967	3.3	5.20	109,979	373.2	591
5	19,282	16,553	523	89,258	387	64,222	154,390	430.8	848	171	118	194	300	783	2.2	4.30	155,173	433.0	853
6	19,613	25,094	2,621	98,971	1,269	272,948	375,809	840.6	2,185	430	474	546	670	2,120	4.7	12.33	377,929	845.3	2,197
7	20,190	18,290	433	87,325	612	76,888	165,258	429.5	1,132	208	134	459	440	1,241	3.2	8.50	166,499	432.7	1,140
8	11,752	13,897	247	41,486	269	62,995	104,997	409.4	719	132	75	294	228	729	2.8	4.99	105,726	412.2	724
9	16,063	15,774	342	61,017	418	61,286	123,063	386.5	1,089	269	124	326	335	1,054	3.3	9.33	124,117	389.9	1,098
10	17,280	17,139	329	79,607	333	83,134	163,403	474.7	1,257	99	125	321	238	783	2.3	6.02	164,186	477.0	1,263
11	23,521	21,919	520	111,131	583	94,172	206,406	454.2	1,564	289	151	638	532	1,610	3.5	12.20	208,016	457.8	1,576
12	21,425	21,732	536	113,262	408	84,888	199,094	461.3	1,486	255	210	479	507	1,451	3.4	10.83	200,545	464.7	1,497
13	6,776	7,278	153	21,982	224	24,480	46,839	333.3	473	104	44	96	112	356	2.5	3.60	47,195	335.8	477
14	12,146	14,831	348	44,796	15,341	183,643	244,128	904.9	2,806	178	78	341	203	800	3.0	9.20	244,928	907.9	2,815
15	13,237	14,303	354	57,563	308	57,058	115,283	418.6	1,441	85	96	361	379	921	3.3	11.51	116,204	421.9	1,453
16	19,961	14,845	521	89,625	416	57,171	147,733	424.4	1,970	374	158	800	592	1,924	5.5	25.65	149,657	430.0	1,995
17	21,755	24,413	605	89,629	587	100,913	191,734	415.3	2,458	430	305	735	563	2,033	4.4	26.06	193,767	419.7	2,484
18	5,984	5,333	109	21,236	73	18,906	40,324	356.3	510	19	18	90	74	201	1.8	2.54	40,525	358.1	513
19	24,368	22,916	705	110,770	665	91,434	203,574	430.5	3,510	302	163	575	634	1,674	3.5	28.86	205,248	434.1	3,539
20	11,659	9,879	247	50,843	285	39,595	90,970	422.4	1,491	172	76	102	149	499	2.3	8.18	91,469	427.7	1,499
21	5,610	4,790	158	27,849	157	20,856	49,020	471.3	1,485	42	105	114	66	327	3.1	9.91	49,347	474.5	1,495
22	11,138	8,802	3,221	86,462	245	39,878	129,806	651.0	3,606	143	73	197	403	816	4.1	22.67	130,622	655.1	3,628
X	13,993	13,998	304	56,795	284	63,535	120,918	432.0	945	77	69	195	169	510	1.8	3.98	121,428	433.8	949
Y	882	495	12	2,347	9	967	3,335	242.2	176	2	0	5	8	15	1.1	0.79	3,350	243.3	176
Total	404,776	402,510	15,157	1,792,501	30,603	2,005,047	3,843,308	476.1	1,356	4,902	3,334	9,268	8,997	26,501	3.3	9.35	3,869,809	479.4	1,365

The ratios for overlapping exons/chromosomal length on chromosomes 22, 19, 14 and 17 were high for 2 exons located on both the same or opposite strands. Ratios were low on chromosomes Y, 13 and 18 for both overlapping types, suggesting that overlapping is not equally distributed among chromosomes. The NIT1/DEDD and ARTS-1/CAST pairs clearly show this overlapping pattern.

## DISCUSSION

Previous reports (5-12) have counted the number of genes exhibiting the overlapping phenomenon, but no reports have enumerated the number of loci or exons that exhibit this overlap. Furthermore, previous reports (5-12) have only described this overlap phenomenon for opposite strands. The present strategy offers a valuable method for estimating the number of overlapping genes, as the total number of genes in the human genome is yet uncertain. Because the total number of genes in the human genome was estimated 32,000 in 2001 (1, 2), and subsequently estimated in 2004 to 22,000.

The total number of exons in the human genome has been estimated at approximately 320,000 (8.8/gene) (1, 2), whereas the present data indicate the existence of more than twice that number. This discrepancy is due to different methods of enumerating exons. We simply counted all of the exons in the human genome, without considering how many exons comprise a gene. This method can identify all exons (e.g., more than 2 exons identified in the same region) and avoids confusion due to splicing variants.

Overlapping genes may evolve as a result of extensions of open reading frames (ORF) caused by switching to an upstream start codon, substitutions in start or stop codons, or deletions and frame shifts that eliminate initiation or stop codons (13). The necessity for maintaining 2 functional overlapping genes inevitably constrains the extent to which both genes can become optimally adapted. However, such constraints can be alleviated by duplication of the overlapping gene pair, allowing for independent evolution of each gene in the resulting copies. This means that overlapping genes can thus only survive long evolutionary periods when the overlap confers a selective advantage upon the organism. In viruses, overlapping genes are thought to persist due to the considerable constraints on genome size (7). In non-viral organisms, the potential advantages of overlapping genes are less clear, although co-regulation may be involved (4). Results of a comparative study of overlapping genes in the genomes of two closely related bacteria revealed that many overlapping genes arise due to incidental elongation of the coding region (16). Overlapping genes have generally been thought to be relatively rare in the human genome, but the results of the present study show that they are more abundant than was previously thought. Interestingly, overlapping genes do not appear to be the result of evolutionary pressure to minimize the size of the human genome.

Yelin *et al*. (17) demonstrated by *in vitro* experiments that antisense transcription occurs widely in the human genome. The resulting data set of 2,667 sense-antisense pairs was evaluated by microarrays containing strand-specific oligonucleotide probes derived from the region of overlap. Verification of specific cases by Northern blot analysis with strand-specific riboprobes confirmed the occurrence of transcription from both DNA strands. While these authors also predicted the existence of approximately 1,600 sense-antisense transcriptional units, transcribed from both DNA strands (13), no overlapping patterns were elucidated.

Adachi-N *et al*. (18) reported that some genes overlap in a head-to-head manner (transcribed in opposite directions), while Koyanagi-KO *et al*. (19) recently reported the occurrence of bidirectional gene pairs in some species. However, they did not describe the patterns of the overlapping exons. In our study, this type of overlap was included in the overlapping loci identified. It has also been reported that divergence (bidirectionality) is frequently observed, particularly in genes involved in DNA repair or replication.(18). The functional significance of this is unclear, but divergence may permit two genes to share one CpG island for purposes of coordinated expression. In some bidirectional loci, expression of two divergent genes has been found to be coregulated, and promoters exhibiting bidirectional activity have often been observed (20, 21). To the best of our knowledge, the phenomenon of overlapping exons is not specific in DNA repair or replication, and further studies are needed to clarify the functional significance of overlapping genes.

Clarification of overlapping genes will facilitate the description of roles for each strand of the human genome and will provide insight into the mechanisms of evolution.

These results show that all overlapping types are distributed throughout the human genome, but that distributions differ for each chromosome.
